# Factors associated with intimate partner violence among ever-partnered women in two Southern African countries: a multilevel analysis

**DOI:** 10.1186/s12905-025-03831-y

**Published:** 2025-07-04

**Authors:** Ololade Julius Baruwa, Elizabeth Biney, Broline Sagini Asuma, Acheampong Yaw Amoateng

**Affiliations:** 1 Centre for Social Science Research, University of Cape Town, Rondebosch, Cape Town South Africa; 2 Ilifa Labantwana, Cape Town, Claremont South Africa; 3 School of Public Health, Catholic University of Ghana, Fiapre Bono Region, Ghana; 4 Maseno University, Maseno, Kenya

**Keywords:** Intimate Partner Violence, Malawi, South Africa, Acceptance wife-beating, Women, DHS

## Abstract

**Background:**

Intimate partner violence (IPV) is a pervasive global health issue with severe physical, emotional, and social consequences for women. In Southern Africa, where IPV rates are high, understanding the factors contributing to IPV is crucial for developing effective interventions. This study examines the factors associated with IPV among ever-partnered women in Malawi and South Africa, two countries with differing socioeconomic contexts but similarly high IPV prevalence.

**Method:**

Data from the 2016 Demographic and Health Survey (DHS) was used, including 7,760 ever-partnered women aged 15–49 from Malawi and South Africa. IPV was defined as lifetime physical, sexual, or emotional violence by a current or former partner and measured as a binary variable (yes/no). The independent variables included age, education, employment, place of residence, wealth status, partner alcohol use, autonomy, inequitable gender attitudes, and IPV history. A generalized linear mixed model (GLMM) was applied to identify IPV-related factors.

**Results:**

Findings revealed that women from Malawi were more likely to experience IPV than women from South Africa (aOR = 2.36, 95% CI = 1.97–2.83; *p* < 0.001). Women aged 25–29 years (aOR = 1.21, 95% CI = 1.03–1.42; *p* = 0.018) and 30–34 years (aOR = 1.30, 95% CI = 1.11–1.52; *p* = 0.001) were more likely to experience IPV than those aged 15–24 years. Women with higher education were less likely to experience IPV (aOR = 0.62, 95% CI = 0.44–0.88; p = 0.007). Employed women were more likely to experience IPV (aOR = 1.13, 95% CI = 1.01–1.26; *p* = 0.037). Women from the rich household wealth index (aOR = 0.81, 95% CI = 0.70–0.93; *p* = 0.003) were less likely to experience IPV than those in the poor wealth index. Women whose partners consumed alcohol (aOR = 2.98, 95% CI = 2.66–3.33; *p* < 0.001), with greater autonomy (aOR = 1.82 95% CI = 1.56–2.12; *p* < 0.001), inequitable gender attitudes (aOR = 1.82, 95% CI = 1.56–2.12; *p* < 0.001), and IPV history (aOR = 1.93, 95% CI = 1.72–2.18; *p* < 0.001) were more at increased risk of IPV.

**Conclusion:**

This study identifies key factors for designing targeted interventions to prevent IPV and improve women’s well-being in Southern Africa.

## Introduction

The act or experience of physical, sexual, and psychological violence by a current or former intimate partner is a major public health and human rights issue worldwide, contributing substantially to the global burden of mental health problems [42, 56]. Available evidence shows that more than a quarter (30%) of women and girls aged 15 and older across the world have experienced physical and/or sexual violence by a close partner [54]. The growing prevalence of intimate partner violence (IPV) is alarming, given its negative health and social implications for individuals, families, and broader society [21]. For the individual, exposure to IPV may result in poor health outcomes, including physical injury, poor mental health, substance abuse, sexually transmitted diseases, unintended pregnancies, and, in extreme cases, death [20, 21, 31]. For society at large, IPV can have a huge negative economic impact due to the direct and hidden costs linked to the provision of medical and other interventions [1, 20].

Although IPV is prevalent in all societies, there are regional and national variations in its levels or degrees owing to sociocultural differences. Comparatively, the incident rate of intimate partner violence in the sub-Saharan African region is the highest in the world, exceeding that of the global rate at a high of 36% [37, 52, 55]. Moreover, the prevalence or incidence of IPV is found to be higher among ever-married or ever-partnered women aged 15 to 49 years [50]. Even within the region, research shows a great deal of variation in IPV prevalence contextually, even though many African countries rank among the highest in world figures [31]. At the southern African level, albeit with rates varying across countries, South Africa and Malawi are among the countries with the highest rates of IPV experienced by women [55].

South Africa boasts staggeringly high rates of IPV despite the country’s progressive human rights ethos and legal regime [20, 53]. For instance, it has been found that half of all South African women murdered are killed by their partners [21, 27], with three women murdered by their partners daily [27]. Even though this data and similar existing figures on IPV cases in South Africa may be conservative estimates, as many incidents go unreported or underdiagnosed.

Although the literature on the prevalence of IPV in Malawi is scarce, the phenomenon is still widespread. However, available research results show growing prevalence and patterns of IPV in Malawi [8, 26, 44]. According to national survey estimates, the 2016 Malawi Demographic and Health Survey (DHS) revealed that about 42% of women had experienced at least one form of IPV (Malawi DHS, 2016) [38].

South Africa and Malawi present good case studies in understanding IPV dynamics and addressing the individual (socio-demographic) and contextual risk factors associated with IPV victimization because of their distinct and diverse cultural, social, and economic landscapes. Malawi is a relatively small, poor, agriculturally landlocked country with strong traditional and patriarchal cultural values, still struggling to modernize and develop within a global capitalist economy [8]. Together with its limited economic and human capital index, many women in Malawi are economically disadvantaged and disproportionally at risk of experiencing IPV. South Africa, on the other hand, is relatively bigger, wealthier, and more advanced, with the largest economy on the continent.

Due to the multidimensional nature of the phenomenon, existing research on intimate partner violence (IPV) reflects the examination of the association between varied socioeconomic factors such as age, socioeconomic status (SES), and substance abuse. For example, studies have found that age and age disparities in relationships are related to the risk of both IPV perpetration and victimization. Many studies have shown that younger women (adolescents and young adults) are particularly at greater risk of IPV victimization compared with older women [5, 24, 33, 46, 49]. Nevertheless, some studies have focused on or highlighted the prevalence of IPV among the elderly, with older women at an increased risk of victimization (see, for example, [36, 57]).

IPV can be better understood using the resource theory developed by Blood and Wolfe [10]. The theory posits that power dynamics within a household are influenced by the resources each member brings. According to the standard resource theory, the partner with more resources—such as income and education—is more likely to be the decision-maker. This power imbalance can significantly influence the prevalence and nature of IPV. Another useful theory that explains IPV is the gendered power theory, developed by Connell [13], which explains how patriarchal structures and unequal power relations between men and women contribute to intimate partner violence (IPV). According to Connell, male dominance within intimate relationships is reinforced by societal norms that privilege men’s authority and control over women. This theory suggests that IPV is a means of maintaining male dominance and reinforcing gender hierarchies.

In general, men with fewer resources (i.e., education, income, and employment) are more likely to be abusive. In comparison, women with fewer resources are more at risk of abuse [15]. In many studies, educational status has consistently been shown to be strongly associated with IPV, with increased educational attainment of both partners or, at the least, of the male partner having a protective effect against IPV [1, 41]. Thus, a lower level of education has been consistently highlighted as a driver of both IPV perpetration and victimisation in different contexts. Accordingly, men with no or low education are more likely to commit violence against their partners [2, 3, 6, 17, 47], whereas women with low educational levels are at higher risk of experiencing violence by their partners [24, 39–41, 58].

Similarly, existing literature regarding the impact of resources on IPV shows that household wealth is protective against IPV as less violence occurs in higher SES groups. Generally, poverty has been identified as a strong contributor to IPV as it increases the risks [11, 19, 47]. In this regard, people living in poverty are disproportionately affected by IPV. Consequently, women who are economically dependent on a male partner are more at risk of abuse and, in most cases, less likely or able to leave abusive relationships [19]. Poorer men (relative to their partners) are more likely to commit violence against their partners [6] IPV against women in Marivan County, Iran.

Also, related to the resource theory, some studies have shown that the employment status of either or both of the couple is significantly related to IPV, with unemployment being a strong predictor of physical violence perpetration and victimization. Regarding the association between male unemployment and IPV, unemployed men are more likely to abuse their partners [3, 4, 11, 24, 25, 41]. Correspondingly, unemployed women are more likely to be abused by their partners [18]. Gainful employment is protective against IPV amongst women in two ways. Firstly, male employment mediates the effects that low income or poverty may have on male-perpetrated IPV. Secondly, female employment provides women with wealth and financial independence from their partners [6].

Place of residence is associated with IPV, with IPV being more prevalent in rural areas than urban areas. Women in rural areas are at a higher risk of IPV, particularly sexual and psychological abuse, compared with women in urban settings [24]. Apart from the usual demographic factors, factors such as excessive alcohol or drug use, acceptance of wife-beating, and a history of abuse have been strongly linked to an elevated risk of women becoming victims of IPV.

Research from multiple studies [2, 6, 16, 17, 25, 28, 41, 47, 49] suggests a correlation between alcohol consumption and male-perpetrated IPV. This correlation is thought to be due to alcohol's known impact on cognitive functioning, leading to increased incidents and severity of partner violence. Consequently, men who misuse alcohol or are addicted to drugs may exhibit a loss of control and aggression towards their partners, contributing to IPV. Similarly, heavy alcohol consumption among women has been linked to increased vulnerability to partner abuse [29].

Research suggests that a history of abuse – either witnessing parental violence in childhood or experiencing abuse as children – is strongly associated with both experiences of perpetration and victimization of IPV [19]. Thus, men who are exposed to violence in childhood are more likely to physically abuse their partners [17, 40, 41, 48]. Equally, women who are exposed to childhood abuse are more likely to experience IPV [25, 49]. The association between childhood exposure to IPV and perpetration and/or victimization could be explained by the fact that exposure increases the probability for individuals to normalize or accept IPV [40].

A growing body of research in sub-Saharan African countries, including Malawi and South Africa, has shown that IPV affects women throughout their lifetime, leading to significant stress, ill health, and negative economic effects [30]. Given the adverse consequences of IPV, it is important to identify key predictors of IPV to design effective and evidence-based interventions and policies to prevent this persistent type of violence. This study uses nationally representative samples to investigate the prevalence and factors associated with IPV among ever-partnered women. In doing so, it contributes to a nuanced understanding of contextual risk factors for IPV victimization.

## Methods

The data for this study was drawn from the most recent Demographic and Health Surveys (DHS) from Malawi and South Africa. The DHS is a nationally representative household survey that measures indicators such as reproductive health, fertility, child and maternal health, HIV/AIDS, nutrition, and mortality across many countries. The DHS uses a stratified two-stage cluster design. The survey used standardized questionnaires, which were similar across countries, yielding comparable data from inter-country. Only one woman per household was chosen for the domestic violence module.

In South Africa, 8514 women were interviewed in the survey, and only 4357 women were selected and interviewed for domestic violence. In Malawi, 24,562 women were successfully interviewed, and only 6379 women were selected and interviewed for domestic violence.

Given that the purpose of this study focused on IPV, the analysis was restricted to 2354 ever-partnered women in South Africa and 5406 ever-partnered women in Malawi who responded to questions on domestic violence. Thus, the unit of analysis for this study was based on 7760 ever-married women who responded to the IPV questions.

## Measures

### Outcome variable

The outcome variable in this study is any form of lifetime IPV, defined as experiencing at least one type of physical, sexual, or emotional violence from a current or former partner. IPV is measured by a “yes” response to any of the following: being pushed, shaken, or having something thrown at her; being slapped; having her arm twisted or hair pulled; being punched or hit with something harmful; being kicked, dragged, or beaten; being choked or intentionally burned; being threatened with a weapon; being humiliated in front of others; being threatened with harm to someone close to her; being insulted or made to feel bad about herself; experiencing unwanted sex; or being forced to have sex.

### Independent variables

The selection of independent variables was informed by previous studies [7, 24, 41]. The independent variables include *age, education, employment, place of residence, wealth status, partner alcohol use, autonomy, inequitable gender attitudes, and IPV history. Age* was defined as women’s current age at the time of the survey and was categorized into six groups: 15–24, 25–29, 30–34, 35–39, 40–44, and 45–49. *Education* referred to the highest level of education completed and was grouped into four categories: no education, primary, secondary and higher. *Employment* was classified as either "working" or "not working." *Place of residence* was categorized as “urban” or “rural,” and the *wealth index* was grouped into three categories: poor, middle, and rich.

*Partner alcohol use* indicated whether the husband or partner consumed alcohol and was measured as "yes" or "no." *Autonomy* was defined based on household decision-making. A respondent was considered to have autonomy if she could independently decide on her own health care, major household purchases, and visiting family or relatives. *Inequitable gender attitudes* were evaluated based on whether participants agreed with at least one reason for a husband to beat his wife. Participants were classified as holding inequitable gender attitudes if they endorsed any of the following reasons: (i) the wife leaves the house without informing her husband, (ii) she neglects the children, (iii) she argues with her husband, (iv) she refuses to have sex with him, or (v) she burns the food. *IPV history* referred to whether the respondent had witnessed her father beating her mother and was measured as "yes" or "no."

## Analysis

This study analyzed data from women aged 15–49 who completed the domestic violence module and had complete information on key intimate partner violence (IPV)-related variables. Exploratory data analysis was conducted to identify and manage missing data. We employed a two-step analytical approach to examine the factors associated with IPV among women. First, descriptive statistics using frequency and percentage distributions were used to summarize the outcome variable (IPV) and the independent variables, including age, education, employment, place of residence, wealth status, partner alcohol use, autonomy, inequitable gender attitudes, and IPV history. Secondly, we applied a generalized linear mixed model (GLMM) to assess the association between the independent variables and IPV. GLMM is a flexible parametric model that accounts for data clustering and correlation within nested structures. In survey data, observations from individuals within the same cluster (e.g., PSU or country) are likely to be correlated, violating the standard regression models' independence assumption. GLMM addresses this by incorporating fixed and random effects to model variability at different hierarchical levels.

For this analysis, we specified a mixed effects model (MEM)**,** including fixed effects (e.g., age, education, employment) and random effects to account for within-cluster correlation. We introduced one random effect to capture clustering at the country level, assuming that participants within the same country share similarities that could influence IPV outcomes. Parameter estimation was conducted using restricted maximum likelihood (REML)**,** which produces unbiased estimates of variance components and robust standard errors. REML is preferred for mixed models as it adjusts for the loss of degrees of freedom due to estimating fixed effects, ensuring more accurate variance component estimates. The model assumes that residuals are normally distributed, the covariance structure is appropriately specified, and that fixed and random effects are correctly incorporated.

The multilevel analysis followed a staged modeling strategy. First, a null model (intercept-only model) was initially fitted to estimate the proportion of variance in IPV explained by the random effect at the country level. Next, Model 1 (Individual-Level Model) included all individual-level predictors such as age, education, employment status, age-disparate relationships, partner’s alcohol intake, autonomy, inequitable gender attitudes and IPV history. Model 2 (Contextual-Level Model) examined household and community-level characteristics, including wealth index, country and place of residence (urban or rural). Finally, Model 3 (Full Model) combined significant individual and contextual-level predictors into a single, comprehensive model.

Survey weights were applied to account for the complex sampling design of the DHS data. The significance of random effects was evaluated using *p*-values at a 95% confidence level. To assess the appropriateness of the multilevel modeling approach, the Intraclass Correlation Coefficient (ICC) was calculated. Model selection and comparison were guided by examining the Akaike Information Criterion (AIC), the Bayesian Information Criterion (BIC), and the Log-Likelihood (LL) values across models. Only adjusted odds ratios (AORs) with 95% confidence intervals were reported in the full model to provide a clearer interpretation of the relationships between predictors and IPV.

## Results

### Characteristics of the study populations

Table 1 presents the proportion of IPV among 7760 ever-partnered women and their demographic characteristics in Malawi and South Africa. Notably, 35.67% of women had experienced some form of IPV from their partners or husbands. Women from Malawi account for the highest proportion (69.20%) of women in the study. A higher proportion of women were under 25 (23.59%). Nearly half (48.03%) had completed primary education, and 42.61% were from poor households. Additionally, 61.05% of the women worked at the time of the survey, and 67.29% lived in rural areas.

**Table 1:** Socio-demographic characteristics and IPV outcome characteristics of ever-partnered women aged 15–49

Variables	n/N	Weighted (95% CI)
**Outcome variables**		
**IPV**		
Yes	2758/7760	35.67 (34.18–37.19)
**Age**		
15–24	1785/7760	23.59 (22.32–24.91)
25–29	1543/7760	20.03 (18.92–21.19)
30–34	1578/7760	20.43 (19.27–21.64)
35–39	1154/7760	14.70 (13.75–15.71)
40–44	934/7760	11.75 (10.87–12.70)
45–49	766/7760	9.50 (8.73–10.32)
**Education**		
No education	845/7760	10.97 (10.09–11.92)
Primary	3754/7760	48.03 (46.33–49.74)
Secondary	2745/7760	34.91 (33.27–36.60)
Higher	416/7760	6.08 (5.18–7.12)
**Employment status**		
Working	4700/7760	61.05 (59.34–62.73)
**Partner’s alcohol intake**		
Yes	2680/7760	33.74 (32.26–35.26)
**Autonomy**		
Yes	2197/7760	29.86 (28.37–31.40)
**Inequitable gender attitudes**		
Yes	933/7760	12.02 (11.01–13.10)
**IPV history (father beats mother)**		
Yes	1801/7760	22.80 (21.52–24.14)
**Wealth index**		
Poor	3203/7760	42.61 (40.77–44.47)
Middle	1613/7760	20.28 (19.04–21.58)
Rich	2944/7760	37.11 (35.16–39.10)
**Place**		
Rural	5355/7760	67.29 (65.34–69.18)
**Country**		
South Africa	2354/7760	30.80 (28.79–32.89)
Malawi	5406/7760	69.20 (67.11–71.21)

Regarding women’s autonomy, 29.86% reported having autonomy in their relationships, and 33.74% stated that their partners drank alcohol, while 12.02% reported Inequitable gender attitudes. Furthermore, 22.8% reported a history of IPV.

#### Multilevel factors associated with IPV: full model

Several factors were associated with IPV in the full model of the logistic regression analysis (see Table 2). Women between the ages of 25–29 years (aOR = 1.21, 95% CI = 1.03–1.42; *p* = 0.018), 30–34 years (aOR = 1.30, 95% CI = 1.11–1.52; *p* = 0.001) and 40–44 years (aOR = 1.22, 95% CI = 1.00–1.49; *p* = 0.017) were more likely to experience IPV compared to women aged 15–24 years. Women with primary education were more likely to experience IPV compared to women with no education (aOR = 1.37, 95% CI = 1.15–1.63; *p* = 0.001). However, women with higher education were less likely to experience IPV than women without education (aOR = 0.62, 95% CI = 0.44–0.88; *p* = 0.007). Employed women were more likely to experience IPV than those not working (aOR = 1.13, 95% CI = 1.01–1.26; *p* = 0.037).

**Table 2:** Multivariable logistic regression analysis of factors associated with IPV among ever-partnered women aged 15–49 in Malawi and South Africa

	**Null model**		**Model I (unadjusted OR)**		**Model II (adjusted OR)**		**Model III (Full model)**	
	**uOR (95% CI)**	***P*-Value**	**aOR (95% CI)**	***P*-Value**	**uOR (95% CI)**	***P*-Value**	**aOR (95% CI)**	***P*-Value**
**Age**								
15–24			1				1	
25–29			1.19 (1.01–1.39)	0.034			1.21 (1.03–1.42)	0.018
30–34			1.26 (1.07–1.48)	0.005			1.30 (1.11–1.52)	0.001
35–39			1.13 (0.95–1.35)	0.181			1.18 (0.98–1.41)	0.076
40–44			1.16 (0.96–1.41)	0.133			1.22 (1.00–1.49)	0.046
45–49			1.05 (0.85–1.30)	0.633			1.11 (0.89–1.37)	0.353
**Education**								
No-education			1				1	
Primary			1.32 (1.11–1.57)	0.002			1.37 (1.15–1.63)	0.001
Secondary			0.88 (0.72–1.08)	0.227			0.96 (0.78–1.18)	0.704
Higher			0.53 (0.38–0.75)	< 0.001			0.62 (0.44–0.88)	0.007
**Employment status**								
Not working			1				1	
Working			1.14 (1.01–1.27)	0.027			1.13 (1.01–1.26)	0.037
**Partner alcohol use**								
No			1				1	
Yes			2.98 (2.67–3.33)	< 0.001			2.98 (2.66–3.33)	< 0.001
**Autonomy**								
No			1				1	
Yes			1.34 (1.19–1.50)	< 0.001			1.59 (1.39–1.82)	< 0.001
**Inequitable gender attitudes**								
No			1				1	< 0.001
Yes			1.81 (1.55–2.11)	< 0.001			1.82 (1.56–2.12)	
**IPV history (father beats mother)**								
No			1				1	
Yes			1.90 (1.69–2.14)	< 0.001			1.93 (1.72–2.18)	< 0.001
**Wealth index**								
Poor					1		1	
Middle					0.85 (0.74–0.97)	0.018	0.89 (0.78–1.03)	0.123
Rich					0.72 (0.64–0.82)	< 0.001	0.81 (0.70–0.93)	0.003
**Place of residence**								
Urban					1		1	
Rura**l**					0.91 (0.78–1.06)	0.216	0.90 (0.77–1.05)	0.162
**Country**								
South Africa					1		1	
Malawi					2.83 (2.44–3.29)	< 0.001	2.36 (1.97–2.83)	< 0.001

Women whose partners consumed alcohol were more likely to experience IPV compared to women whose partners do not drink alcohol (aOR = 2.98, 95% CI = 2.66–3.33; *p* < 0.001). Women with autonomy were more likely to experience IPV compared to women with no autonomy (aOR = 1.59, 95% CI = 1.39–1.82; *p* < 0.001). Women with inequitable gender attitudes were more likely to experience IPV compared with women with equitable attitudes (aOR = 1.82, 95% CI = 1.56–2.12; *p* < 0.001). Women who reported IPV history were two times more likely to experience IPV compared with women with no IPV history (aOR = 1.93, 95% CI = 1.72–2.18; *p* < 0.001). Women from the rich wealth index (aOR = 0.81, 95% CI = 0.70–0.93; *p* = 0.003) were less likely to experience IPV than those in the poor wealth index. Lastly, women from Malawi were more likely to experience IPV compared to women from South Africa (aOR = 2.12, 95% CI = 1.76–2.54; *p* < 0.001).

#### Random effect analysis and model fitness comparison

The null model revealed that 14.5% of the variation in IPV was attributable to differences between clusters, with a cluster-level variance of 0.32 (95% CI: 0.22–0.43), highlighting significant contextual influences and justifying the use of a multilevel model (see Table 3). As explanatory variables were introduced across Models 1 to 3, both the cluster-level variance and intraclass correlation coefficient (ICC) declined, indicating that the included individual- and contextual-level covariates explained a meaningful portion of the between-cluster differences. Concurrently, model fit improved, as evidenced by lower AIC values and higher log-likelihoods, confirming that the addition of covariates enhanced the models' explanatory power. Despite this, some residual cluster-level variation persisted, suggesting that unmeasured community-level factors may still contribute to IPV risk.

**Table 3:** Variance estimates, intra-class correlation coefficient and model fit test for the multilevel models of IPV among ever-partnered women aged 15–49 in Malawi and South Africa

Random Effect Parameters	Null	Model 1	Model 2	Model 3
Cluster-level variance (PSU) (95% CI)	0.32 (0.22–0.43)	0.23 (0.15–0.35)	0.32 (0.23–0.44)	0.24 (0.16–0.36)
ICC (%)	14.5%	10.6%	8.8%	6.7%
**Model fit statistics**				
Akaike Information Criterion (AIC)	9765.81	9087.91	9734.95	9095.55
Bayesian Information Criterion (BIC)	9786.68	9199.22	9776.69	9220.77
Log-Likelihood (LL)	-4879.91	-4527.96	-4861.47	-4429.78
Number of clusters	1511	1511	1511	1511

*CI* Confidence Interval, *PSU* Primary sampling unit, *ICC* Intra-class correlation coefficient

## Discussion

The present study sought to examine the predictors of IPV in Malawi and South Africa using nationally representative data from the 2016 Malawi and South Africa DHS. The study revealed that at least one in every three women had experienced some form of IPV from their partners or husbands. The findings also demonstrate how factors such as age, socio-economic status (education, employment and wealth index), cultural norms (inequitable gender attitudes), partner alcohol use, autonomy, and IPV history intersect to exacerbate women’s vulnerability to IPV victimization.

Our analysis revealed that women in Malawi were more likely to experience IPV than their South African counterparts. This finding aligns with existing research showing that approximately 20% of Malawian women have experienced physical IPV, with 13% facing emotional and sexual abuse [14]. In contrast, while IPV remains a significant issue in South Africa, our findings suggest that the overall risk is relatively lower. This finding may be linked to South Africa's stronger legal protections, higher levels of gender equality, and greater access to support services, which may mitigate some structural vulnerabilities that increase IPV risk in Malawi [26].

Intersectionality theory helps explain these disparities by highlighting how intersecting social factors influence IPV risk. Intersectionality theory, introduced by Crenshaw [12], explains how overlapping social identities — such as gender, race, class, and geographic location — create unique experiences of oppression and vulnerability. First, socioeconomic status plays a significant role,higher poverty rates in Malawi may exacerbate IPV risk by increasing women's economic dependence on male partners, limiting their ability to leave abusive relationships [22]. Secondly, legal and policy framework differences may contribute to the variation in IPV rates. South Africa has implemented comprehensive legal measures to combat gender-based violence, while enforcement and access to justice may be weaker in Malawi. Finally, cultural norms around gender roles and male dominance also shape IPV risk. Patriarchal norms that justify male control over women may be more deeply entrenched in Malawi, reinforcing the power imbalance that increases vulnerability to IPV [25].

Our findings that women with lower educational attainment and financial constraints are at higher risk of IPV align with resource theory, which posits that unequal access to resources can create power imbalances, increasing the likelihood of IPV. The multivariate analysis indicated that women with better education (higher) were less likely to experience IPV, corroborating with previous studies [2, 3]). Education is often linked to greater agency and empowerment. Higher education levels can help women make informed decisions and be more aware of their rights, which can act as barriers against IPV. Educated women are more likely to be aware of social issues, legal rights, and support networks, enabling them to take proactive steps to escape or avoid abusive environments. Additionally, higher education is associated with better economic prospects, leading to financial independence and reducing reliance on an abusive partner.

Financial strain or economic pressures can add to relationship stress, particularly if the woman is the main provider. According to resource theory [10], disparities in wealth between partners can lead to power struggles and imbalances, increasing the risk of IPV. Women’s wealth index was also associated with IPV in this study. Specifically, the prevalence of IPV was lower among women in the high wealth index. Poverty-related stressors can exacerbate the risk of abuse and violence in relationships. Women in the poor wealth index may have less power and autonomy in household decision-making [34]. Conversely, women from the rich wealth index may underreport their IPV experiences due to social desirability and family prestige. This finding is consistent with previous studies [6, 11, 19, 25, 47], which reported that women in the poor and middle wealth index were more likely to report IPV compared to women from the rich wealth index.

Unlike previous studies [3, 18, 24], this study found that employed women were more likely to experience IPV. Employment can shift power dynamics within the household, challenging traditional gender roles and potentially making partners feel threatened, particularly when the woman is the sole earner or earns more than her partner [50]. Additionally, the demands and stress of employment, especially in high-pressure work environments, can heighten tension and conflict at home, thereby increasing the risk of IPV [50]. Similar to the association between employment and IPV found in our study, and contrary to expectations, this study also revealed that women's autonomy increases the risk of experiencing IPV. While it is generally assumed that greater autonomy would reduce the risk of IPV [9, 35], our findings suggest the opposite. Women with higher autonomy may face increased IPV because their independence and decision-making power can challenge traditional gender norms, where men are viewed as the primary providers and decision-makers. This disruption of traditional roles may create tension and conflict, leading to IPV [45, 51], underscoring potential cultural differences in how women’s autonomy is perceived and its impact on IPV.

The association between IPV, inequitable gender attitudes, and partner alcohol consumption reflects deep-rooted patriarchal norms and unequal power dynamics within relationships. Gendered power theory explains how these norms reinforce male dominance and control, increasing women’s vulnerability to IPV. A history of violence and harmful cultural norms, such as inequitable gender attitudes, often perpetuate gender inequalities and exacerbate power imbalances in relationships [10, 16, 28]. Women who internalize these harmful norms and practices are more likely to experience IPV. The present study found that women whose partners drank alcohol faced a higher risk of IPV, consistent with findings from previous studies in Nepal [6], Nigeria [41], and Asia and the Pacific [25]. This finding may be due to alcohol’s disinhibitory effects or its use by partners to lower inhibitions and engage in undesirable behaviours, including sexual and physical violence [32].

Women who have a history of IPV in their family (i.e., whose father has ever abused their mother) were found to be at risk of IPV. This finding aligns with a previous study in South Africa, which reported that exposure to violence in childhood is a strong predictor of IPV [19]. Moreover, our finding is consistent with a cross-sectional study across nine countries, which reported a significant association between experiencing IPV and witnessing one’s mother being hit by a partner in six out of the nine countries analyzed [49]. Similarly, a study in Sao Paulo, Brazil, found that women who reported that their mother was hit by their father experienced excessive IPV [29]. These results suggest that children might model the behaviour of their parents, leading to a cycle of violence where individuals replicate the behaviours they witnessed, perpetuating IPV across generations. Witnessing IPV during childhood can lead to low self-esteem, powerlessness, or insecure attachment, which may prevent women from having healthy relationships.

Lastly, the age of women was observed to influence IPV significantly. Specifically, women aged 25–34 years were more prone to experiencing IPV. This could be attributed to the fact that women in this age group are often in longer-term relationships, such as marriage or cohabitation, which may involve relationship stressors that make them more susceptible to IPV [43]. Additionally, this age group frequently undergoes significant life transitions, such as starting families and taking on more responsibilities, children which can increase relationship stress and the risk of IPV.

### Study limitations

The cross-sectional design restricts our ability to conclude causality, as it captures data at a single point, making it difficult to establish the temporal order of events. Furthermore, given the sensitivity of the issue (IPV), there is a potential for under-reporting and recall bias, as respondents may either forget details or feel uncomfortable sharing sensitive information. However, the DHS program implemented measures to mitigate these challenges, including specialized training for fieldworkers on data collection, ensuring privacy during interviews, and emphasizing confidentiality to encourage honest responses. While these limitations should be considered when interpreting the findings, the study still provides valuable and representative insights into the social and demographic contexts of the issue, contributing to the broader body of literature in this field.

## Conclusion

The findings from the present study highlight that IPV is closely linked to cultural norms like inequitable gender attitudes, women's autonomy, partner's alcohol use, a history of IPV, and various socioeconomic factors, including age, education, employment, and wealth index. Policymakers should implement educational programs and media campaigns to address harmful cultural norms and promote gender equality. In addition, policymakers should engage communities in dialogues to dismantle patriarchal structures and increase IPV awareness via public service announcements and outreach programs. Both Malawi and South Africa need to strengthen policies to reduce alcohol abuse among men, including rehabilitation services and awareness programs. Interventions to break the cycle of IPV should offer counseling for individuals with a history of family violence and integrate IPV education into school curriculums. Further, policy should target support for women's autonomy through gender-sensitivity training and equitable household decision-making and workplace stressors.

## Data Availability

The study used secondary data from dhsprogram.org, which is publicly available at https://dhsprogram.com/data/available-datasets.cfm.
